# C-terminal truncated hepatitis B virus X protein promotes hepatocellular carcinogenesis through induction of cancer and stem cell-like properties

**DOI:** 10.18632/oncotarget.8209

**Published:** 2016-03-19

**Authors:** Kai-Yu Ng, Stella Chai, Man Tong, Xin-Yuan Guan, Chi-Ho Lin, Yick-Pang Ching, Dan Xie, Alfred Sze-Lok Cheng, Stephanie Ma

**Affiliations:** ^1^ School of Biomedical Sciences, Li Ka Shing Faculty of Medicine, The University of Hong Kong, Pok Fu Lam, Hong Kong; ^2^ Department of Clinical Oncology, Li Ka Shing Faculty of Medicine, The University of Hong Kong, Pok Fu Lam, Hong Kong; ^3^ State Key Laboratory for Liver Research, Li Ka Shing Faculty of Medicine, The University of Hong Kong, Pok Fu Lam, Hong Kong; ^4^ Centre for Genomic Sciences, Li Ka Shing Faculty of Medicine, The University of Hong Kong, Pok Fu Lam, Hong Kong; ^5^ State Key Laboratory of Oncology in Southern China, Sun Yat-Sen University Cancer Center, Guangzhou, China; ^6^ School of Biomedical Sciences, The Chinese University of Hong Kong, Sha Tin, Hong Kong

**Keywords:** cancer stem cells, HBx, HCC, tumor-initiating cells, RNA-Seq

## Abstract

Tumor relapse after chemotherapy typifies hepatocellular carcinoma (HCC) and is believed to be attributable to residual cancer stem cells (CSCs) that survive initial treatment. Chronic infection with hepatitis B virus (HBV) has long been linked to the development of HCC. Upon infection, random HBV genome integration can lead to truncation of hepatitis B virus X (HBx) protein at the C-terminus. The resulting C-terminal-truncated HBx (HBx-ΔC) was previously shown to confer enhanced invasiveness and diminished apoptotic response in HCC cells. Here, we found HBx-ΔC to promote the appearance of a CD133 liver CSC subset and confer cancer and stem cell-like features in HCC. HBx-ΔC was exclusively detected in HCC cell lines that were raised from patients presented with a HBV background with concomitant CD133 expression. Stable overexpression of the naturally occurring HBx-ΔC mutants, HBx-Δ14 or HBx-Δ35, in HCC cells Huh7 and immortalized normal liver cells MIHA resulted in a significant increase in the cells ability to self-renew, resist chemotherapy and targeted therapy, migrate and induce angiogenesis. MIHA cells with the mutants stably overexpressed also resulted in the induction of CD133, mediated through STAT3 activation. RNA sequencing profiling of MIHA cells with or without HBx-ΔC mutants stably overexpressed identified altered FXR activation. This, together with rescue experiments using a selective FXR inhibitor suggested that C-terminal truncated HBx can mediate cancer stemness via FXR activation. Collectively, we find C-terminal truncated HBx mutants to confer cancer and stem cell-like features *in vitro* and to play an important role in driving tumor relapse in HCC.

## INTRODUCTION

Chronic hepatitis B virus (HBV) infection is a major risk factor in the development of hepatocellular carcinoma (HCC) in Southeast Asia. Approximately 90% of HCC is due to HBV infection in the locality. Epidemiological studies have shown that the relative risk of HCC among HBV carriers is 10-fold higher than that of non-carriers [1]. Thus, an understanding of the mechanism by which HBV induces HCC formation is of great significance. HBV DNA is integrated and often highly rearranged within the host DNA in HCC tumors [2–3]. These templates frequently produce a 154-amino acid HBV viral oncoprotein, HBx, which is active in transactivation assays [4]. The sustained production of HBx is associated with hepatocellular transformation and represents a major contribution of HBV to HCC [5]. We and others have reported that HBV integration is detected in 80–90% of host genomes from HBV-infected HCC cases and that the HBx gene is often partially deleted during the integration process, causing the C-terminal truncation of HBx [6–9]. We and others have also found, through both *in vitro* and *in vivo* studies, that C-terminal truncated HBx (HBx-ΔC) plays a critical pro-oncogenic and pro-metastatic role in hepatocarcinogenesis [10–13]. A recent study by Quetier et al. found that the HBx protein with C-terminal deletions was more susceptible to DEN-induced hepatocarcingoenesis than the full-length HBx protein in a mice model, through increased expression of IL-6, TNF-α and IL-1β transcripts as well as activation of STAT3, ERK and JNK proteins [14]. These results demonstrate that, in addition to the full-length HBx, HBx-ΔC also plays an important, and likely a more critical role, in HCC development.

Recent compelling evidence has emerged in support of a cancer stem cell (CSC)/tumor-initiating cell (T-IC) model in leukemia and a wide range of solid tumors, including HCC. CSCs are believed to harbor both cancer cell- and stem cell-like characteristics, including uncontrolled growth, self-renewal, differentiation and chemoresistance. These cells are now widely regarded as the root of tumor origin and recurrence. In HCC, specifically, microarray analyses of human HCC samples identified the molecular similarities between CSCs and hepatic stem cells highlighted the importance of CSCs in the progression of the disease [15]. We and others have identified important functionally defined liver CSC subsets that is marked phenotypically by CD133 and aldehyde dehydrogenase (ALDH) activity [16–20]. Liver CSC subsets that are positive for CD133 and ALDH possess preferential abilities to self-renew, differentiate, initiate tumors and resist chemotherapy [16–17, 20–22]. CD133^+^ cells also have prognostic value in HCC and play an important functional role in regulating tumorigenesis. Despite our growing understanding of the importance and existence of such liver CSC subpopulations, the mechanism by which these cells are activated in HCC remains elusive. Two recent studies have found that full-length HBx can induce stem cell-like and CSC-like signatures in HCC in human [23] and mouse models [24]. However, the role of HBx-ΔC in induction of “stemness” phenotypic properties and induction of liver CSC subsets has not been explored.

In this study, we tested the hypothesis that the frequent carboxyl-terminal truncated from of HBx contributes to hepatocarcinogenesis through the induction of cancer and stem cell-like properties. Specifically, HBxΔC14 and HBxΔC35 were chosen for studies because these C-terminal truncated HBx variants have previously been shown to abrogate the growth suppressive effects induced by full-length HBx, and as a result, can effectively promote cell transformation and enhance the proliferative activity of neoplastic cells [8, 11, 25]. More importantly, they have been identified as natural deletion mutants in HCC tissues [8, 11, 25]. We found these two HBx-ΔC mutants to promote the appearance of a CD133 liver cancer stem cell subset and confer cancer and stem cell-like properties in HCC cell line models. HBx-ΔC was exclusively detected in HCC cell lines that were raised from patients presented with a HBV background with concomitant CD133 expression (Hep3B, PLC8024, SNU182 and SNU475). The stable overexpression of HBx-Δ14 or HBx-Δ35 in HCC cell line Huh7 and immortalized normal liver cell line MIHA resulted in a significant increase of the cells ability to self-renew, resist 5-fluorouracil and sorafenib, migrate and induce capillary tube formation in endothelial cells. Overexpression of the two naturally occurring C-terminal truncated mutants in MIHA cells also resulted in an increase in the CD133 subset, mediated by STAT3 activation. RNA sequencing profiling of MIHA cells with or without HBx-ΔC stably overexpressed, combined with subsequent rescue experiments using a specific FXR inhibitor, identified altered FXR activation in C-terminal truncated HBx to play a critical role in conferring cancer and stemness properties *in vitro*.

## RESULTS

### Presence of HBV genome in HCC is associated with increased stemness properties

**Figure 1:**
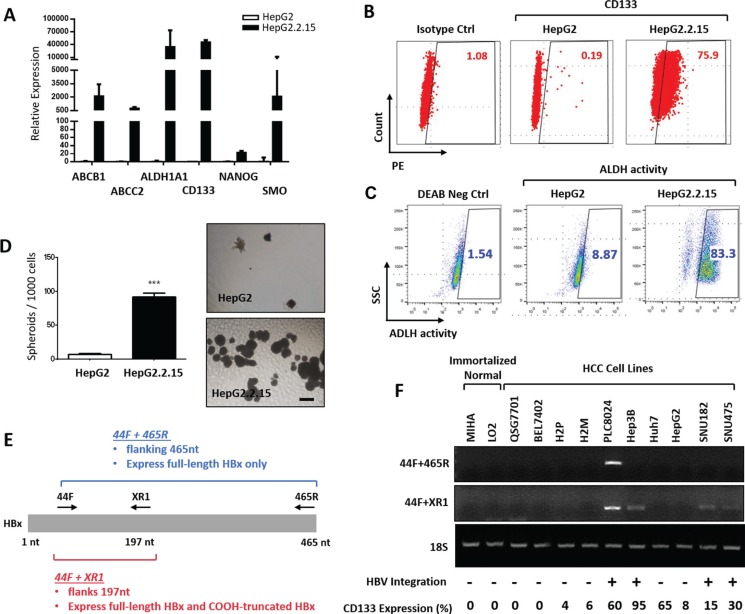
(**A**) Relative expression of stemness associated genes in HepG2 and HepG2.2.15 HCC cells. (**B**) Flow cytometry dot plot analysis for CD133 expression in HepG2 and HepG2.2.15 HCC cells. (**C**) Flow cytometry analysis for ALDH activity using the ALDEFLUOR kit in HepG2 and HepG2.2.15 cells. DEAB stands for negative control when cells were treated with an ALDH inhibitor, diethylaminobenzaldehyde. (**D**) Representative image and quantification of hepatospheres generated from HepG2 and HepG2.2.15 HCC cells. Scale bar = 100 μm. ***p < 0.001. (**E**) Detection of full-length and C-terminal truncated HBx by RT-PCR using two sets of primers. 44F and XR1 flanks 197 nucleotides. 44F and 465R flanks 465 nucleotides. (**F**) RT-PCR analysis of full-length and C-terminal truncated HBx in a panel of immortalized normal and HCC cell lines. 18S was amplified as an internal control. CD133 expression was determined by flow cytometry analysis.

HepG2.2.15 cells are derived from the human hepatoblastoma cell line HepG2 and are characterized by having stable HBV expression and replication in the culture system [26]. As compared to parental HepG2 cells, we found HepG2.2.15 cells to preferentially express stemness associated genes including NANOG, SMO, ABCB1, ABCC2, aldehyde dehydrogenase 1A1 (ALDH1A1) as well as the functional liver CSC marker CD133, as detected by qRT-PCR (Figure 1A). Enhanced expression of CD133 (0.19% to 75.9%) and ALDH activity (8.87% to 83.3%) in HepG2.2.15 was further confirmed by flow cytometry analyses (Figure 1A–1B). In addition, HepG2.2.15 also displayed an enhanced ability to form hepatospheres *in vitro*, suggesting the presence of self-renewing cells (Figure 1D). This suggests that the presence of an HBV genome is associated with stemness properties in HCC.

### C-terminal truncated HBx is correlated with CD133 expression in HCC cell lines

Previous work by us and others have found both full-length and C-terminal truncated HBx to play critical pro-oncogenic and pro-metastatic roles in hepatocarcinogenesis [10–14, 27–29]. Two recent studies have also found full-length HBx to induce stem cell-like and CSC-like signatures in HCC [23–24]. We set out to examine the correlation between the expression of full-length, C-terminal truncated HBx and the well-document functional liver CSC marker CD133 in a panel of immortalized normal liver (MIHA and LO2) and HCC cell lines (QSG7701, BEL7402, H2P, H2M, PLC8024, Hep3B, Huh7, HepG2, SNU182 and SNU475) in the absence or presence of HBV background by qRT-PCR and flow cytometry. Specifically, for PCR amplification of HBx, sets of PCR primers (44F and XR1 or 44F and 465R) were used for full-length and C-terminal truncated HBx, respectively (Figure 1E). Clones that can only be amplified by the primers for the smaller fragment (44F and XR1; flanking 197nt), but not for the full-length fragment (44F and 465R; flanking 465nt), was considered positive for C-terminal truncated HBx (HBx-ΔC). Of the cell lines that expressed some degree of CD133 expression, PLC8024 has previously been shown to contain multiple copies of the integrated HBV genome [30] while Hep3B contains only a single copy of integrated HBV genome [31]. Huh7 and HepG2 cells were obtained from patients with no history of HBV infection [32]; while both SNU182 and SNU475 are known to have HBV integration in their genome [33]. H2P and H2M were derived from a HCC patient that was negative from serologic HBV [34]. A positive correlation between C-terminal truncated HBx and CD133 expression was observed across this panel of cell lines. HCC cell lines that contain HBV integration in their genome (PLC8024, Hep3B, SNU182 and SNU475) and express CD133 were also found to contain the C-terminal truncated HBx DNA fragment. HCC cell lines that were raised from patients without HBV infection, but express CD133 (H2P, H2M, Huh7 and HepG2), were not found to contain the C-terminal truncated HBx DNA fragment. Immortalized normal liver cell lines (MIHA and LO2) and other HCC cell lines that were raised from patients without HBV infection and do not express CD133 (QSGY7701, BEL7402) were also absent for C-terminal truncated HBx (Figure 1F). Conversely, full-length HBx was only detected in PLC8024 and did not correlate with CD133 expression in HCC (Figure 1F).

### C-terminal truncated HBx overexpression induced cancer and stem cell-like properties in HCC cell lines

**Figure 2:**
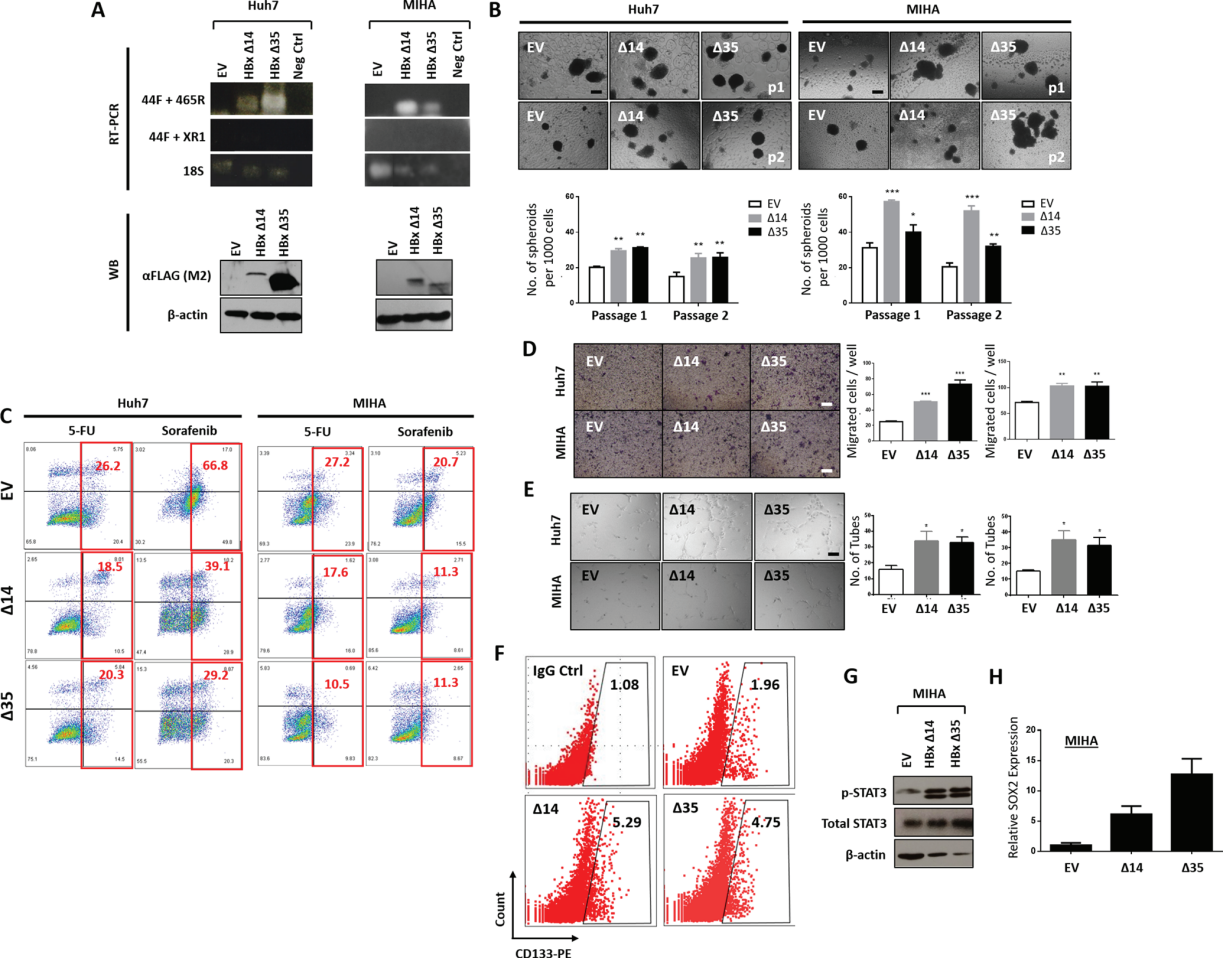
(**A**) Validation of HBx-Δ14 and HBx-Δ35 (expressing 14- and 35-amino acid C-terminal truncation) overexpression into HBV negative, CD133 absent MIHA cells and HBV negative, CD133 present Huh7 cells at genomic levels by RT-PCR and proteomic levels by Western blot. Empty vector (EV) transfected as control. 18S and beta-actin as internal controls for RT-PCR and Western blot, respectively. (**B**) Representative image and quantification of hepatospheres (primary and secondary passages) in MIHA or Huh7 cells with HBx-Δ14 and HBx-Δ35 stably overexpressed. Scale bar = 100 μm. ***p < 0.001, **p < 0.01, *p < 0.05. (**C**) Percentage of Annexin V positive cells in MIHA or Huh7 cells with HBx-Δ14 and HBx-Δ35 stably overexpressed, following 5-fluorouracil (5-FU) or sorafenib treatment. (**D**) Representative image and quantification of number of cells that migrated in MIHA or Huh7 cells with HBx-Δ14 and HBx-Δ35 stably overexpressed. Scale bar = 100 μm. ***p < 0.001, **p < 0.01. (**E**) Representative image and quantification of capillary tubes formed by HUVECs following treatment with supernatant collected from MIHA or Huh7 cells with HBx-Δ14 and HBx-Δ35 stably overexpressed. Scale bar = 100 μm. *p < 0.05. (**F**) Flow cytometry dot plot analysis for CD133 expression in MIHA cells with HBx-Δ14 and HBx-Δ35 stably overexpressed. (**G**) Western blot analysis of MIHA with EV, HBx-Δ14 or HBx-Δ35 stably overexpressed for phosphorylated and total STAT3 expression. (**H**) Relative expression of SOX2 in MIHA cells with or without HBx-Δ14 and HBx-Δ35 stably overexpressed.

In view of the close association between C-terminal truncated HBx and CD133, we then chose to focus our phenotypic characteristics of HBx-associated “stemness” in the C-terminal truncated mutants only. Stable overexpression of HBxΔC14 and HBxΔC35 was established in HBV negative, CD133 absent MIHA cells and HBV negative, CD133 positive Huh7 cells. RT-PCR and Western Blot analyses were performed to validate overexpression of the two C-terminal truncated HBx variants at the genomic and proteomic levels, respectively (Figure 2A).

To delineate the functional relevance of the observed positive correlation between natural C-terminal truncated HBx and CD133 in HCC cell lines, we performed various *in vitro* assays to examine the ability of C-terminal truncated HBx to modify cancer and stem cell properties, including their abilities to form spheres and serially passage in 3D culture systems (clonogenic potential), to resist chemotherapy and targeted therapy treatment, as well as to promote cell migration and capillary tube formation in endothelial cells. Stable overexpression of HBxΔC14 and HBxΔC35 in both MIHA and Huh7 led to an enhanced ability of the cells to form bigger and more hepatospheres in both primary and secondary passages in a significantly shorter period of time, than as compared to EV controls (Figure 2B). HCC cells with HBxΔC14 and HBxΔC35 stably overexpressed were also less sensitive to chemotherapy 5-fluorouracil and targeted therapy sorafenib, as evident by the significant decrease in apoptotic / necrotic cells. 5-fluorouracil and sorafenib induced apoptosis in MIHA cells decreased from 27.2% to 17.6% and 10.5% and 20.7% to 11.3% and 11.3%, respectively. Similar phenomenon was also observed in Huh7 cells where percentage of dead cells following the two treatment regimens decreased from 26.2% to 18.5% and 20.3% and 66.8% to 39.1% and 29.2%, respectively (Figure 2C). Further, stable overexpression of HBxΔC14 and HBxΔC35 also led to a significant increase in the ability of HCC cells to migrate (Figure 2D). Human umbilical vein endothelial cells (HUVEC) treated with conditioned media collected from HBxΔC14 and HBxΔC35 overexpressed HCC cells showed an enhanced ability to induce capillary tube formation as compared with medium collected from EV control cells (Figure 2E). Following stable overexpression of the two truncated HBx variants in CD133 absent/low MIHA cells, expression of CD133 also increased by over 2 to 3 folds, from 1.96% (EV) to 5.29% (HBxΔC14) and 4.75% (HBxΔC35). (Figure 2F). Note that since CD133 expression is already expressed at very high levels in Huh7, CD133 expression was not found to be altered following truncated HBx variants overexpression (data not shown). A recent publication by our collaborator Won et al. found STAT3 to promote CD133 transcription [35]. Consistent with this, we also found enhanced CD133 expression in the two HBx variants to also concomitantly express elevated p-STAT3 in MIHA cells, suggestive that p-STAT3 represents the mediator or initial target molecule for CD133 transcription activation (Figure 2G). In addition, a marked up-regulation of SOX2 expression was also observed following overexpression of the two C-terminal truncated mutants in MIHA cells (Figure 2H).

### Transcriptome sequencing profiling identifies unique gene signatures and functional network relating to farnesoid X receptor (FXR) to be preferentially deregulated in C-terminal truncated HBx driven HCC

**Figure 3:**
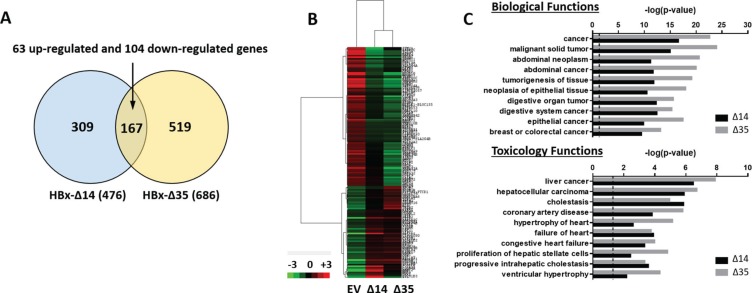
(**A**) Venn diagram of differentially regulated genes based on gene fold change ≥ 1.5 and FDR ≤ 0.05 between EV vs. HBx-Δ14 and EV vs. HBx-Δ35 in MIHA cells. Genes that were significantly and commonly deregulated are shown in the overlapping area. (**B**) Unique gene signatures of MIHA cells with or without expression of HBx-Δ14 and HBx-Δ35, as shown by hierarchical cluster analysis (fold change ≥ 1.5 and FDR ≤ 0.05). Each cell in the matrix represents a particular expression level, where red and green cells indicate high and low gene expression, respectively. (**C**) Functional analysis of C-terminal truncated HBx mutants regulated genes by IPA. The *p* value was calculated using Fisher exact test to show the likelihood of association between our dataset of deregulated genes and a biological or toxicological function.

**Figure 4:**
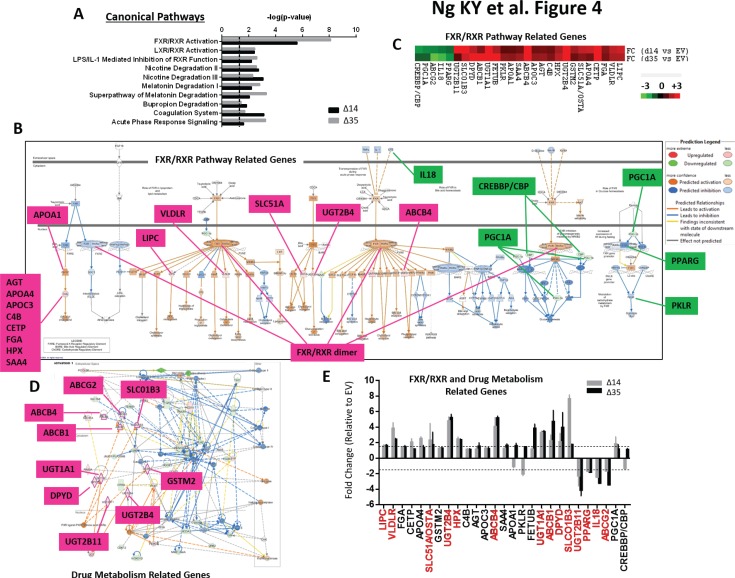
(**A**) Pathway analysis of C-terminal truncated HBx mutants regulated genes by IPA. The *p* value was calculated using Fisher exact test to show the likelihood of association between our dataset of deregulated genes and canonical pathways. (**B**) FXR/RXR interaction and functional network identified by IPA. Genes in red and green indicate up- and down-regulated genes found in C-terminal truncated HBx mutants, respectively. Genes in white are not included in our dataset, but added by IPA to complete the network connections. Molecules predicted to be activated and inhibited by the IPA molecule activity predictor are labeled in orange and blue, respectively. (**C**) Heatmap showing fold change of individual genes involved in FXR/RXR pathway and drug metabolism. A total of 22 and 5 genes were found commonly up- and down-regulated, respectively. Each cell in the matrix represents a particular expression level of genes, where red and green cells indicate high and low gene expression, respectively. (**D**) Drug metabolism interaction and functional network identified by IPA. Genes in red and green indicate up- and down-regulated genes found in C-terminal truncated HBx mutants, respectively. Genes in pink are related to drug metabolism. Genes in white are not included in our dataset, but added by IPA to complete the network connections. Molecules predicted to be activated and inhibited by the IPA molecule activity predictor are labeled in orange and blue, respectively. (**E**) qRT-PCR validation of identified differentially expressed genes relating to FXR/RXR pathway and drug metabolism in MIHA cells with or without HBx-Δ14 and HBx-Δ35 stably overexpressed.

**Figure 5:**
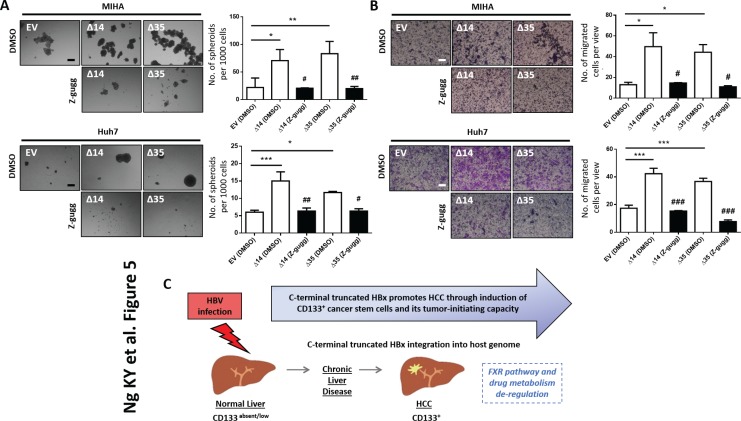
(**A**) Representative image and quantification of hepatospheres in MIHA cells with HBx-Δ14 and HBx-Δ35 stably overexpressed, in the absence or presence of FXR inhibitor, Z-guggulsterone (Z-gugg; 25 μM). Scale bar = 100 μm. *compared to EV with ***p < 0.001, **p < 0.01, *p < 0.05. ^#^compared to DMSO with ^##^*p* < 0.01, ^#^*p* < 0.05. (**B**) Representative image and quantification of number of cells that migrated in MIHA cells with HBx-Δ14 and HBx-Δ35 stably overexpressed in the absence or presence of FXR inhibitor, Z-guggulsterone (Z-gugg; 25 μM). Scale bar = 100 μm. *compared to EV with ***p < 0.001, *p < 0.05. ^#^compared to DMSO with ^###^*p* < 0.001, ^#^*p* < 0.05. (**C**) Cartoon summary of the role of C-terminal truncated HBx variants, in particular HBx-Δ14 and HBx-Δ35, in promoting cancer and stem cell-like features *in vitro* in HCC.

In an attempt to characterize the molecular mechanisms by which C-terminal truncated HBx mutants mediate cancer properties, self-renewal and resistance to therapy, RNA-Seq profiling was employed to compare gene expression profiles between control and C-terminal truncated HBx mutants isolated from MIHA cells. Following filtering, ~97% of the reads were used for downstream analysis. Of this, ~76% of the reads mapped to the human transcriptome reference (hg19). Expression levels were tabulated in accordance with the number of transcripts per million (TPM). At a 1.5-fold cut-off with a FDR ≤ 0.05, a total of 995 genes were found de-regulated, of which 167 genes were found commonly differentially expressed between the two C-terminal truncated HBx variants (63 up-regulated and 104 down-regulated) (Figure 3A). A visualization of the differential expression pattern for these 167 genes is shown in Figure 3B using a hierarchical clustering heat map (Figure 3B). To study biological functions, Gene Ontology (GO) analysis was performed on the de-regulated genes with FDR ≤ 0.05. A selection of the significant GO terms for biological and toxicity functions is shown in Figure 3C (*p* ≤ 0.05 and –log (*p* value) ≥ 1.3), with most of the terms closely relating to cancer and in particular, liver cancer and hepatocellular carcinoma (Figure 3C). The same gene set was also surveyed using the pathway analysis tool by Ingenuity Pathway Analysis (IPA) where FXR/RXR activation (Figure 4A–4C) (*p* ≤ 0.05 and –log (*p* value) ≥ 1.3) and interactions between drug metabolism related genes (Figure 4B) were found well represented in the de-regulated gene set (Figure 4A–4C). FXR/RXR pathway activation and drug metabolism related genes have previously been shown to play critical roles in mediating chemoresistance, and in particular liver tumor chemoresistance against genotoxic compounds [36–38]. To validate our profiling results, qRT-PCR was performed to validate 27 selected commonly deregulated mRNAs involved in FXR/RXR activation and drug metabolism, including LIPC, VLDLR, FGA, CETP, APOA4, SLC51A/OSTA, GSTM2, UGT2B4, HPX, C4B, AGT, APOC3, ABCB4, SAA4, APOA1, PKLR, FETUB, UGT1A1, ABCB1, DPYD, SLCO1B3, UGT2B11, PPARG, IL18, ABCG2, PGC1A, CREBBP/CBP, of which over half could be confirmed in both C-terminal truncated mutants (Figure 4E, highlighted in red), suggesting that C-terminal truncated HBx mutants confer resistance to standard therapy via activation of these predicted pathways. To further confirm the involvement of FXR pathway activation, a selective FXR inhibitor (Z-guggulsterone) was used as a rescue in several *in vitro* functional studies. Addition of Z-guggulsterone (25 μM) in C-terminal truncated HBx mutants attenuated the abilities of the cells to form hepatospheres (Figure 5A) and migrate (Figure 5B), suggesting that FXR activation in C-terminal truncated HBx mutants is critical in conferring cancer and stemness properties to these cells. Note that rescue experiments were also carried out using a selective RXR inhibitor (UVI 3003). However, its addition did not result in a rescued functional phenotype (data not shown).

## DISCUSSION

Chronic hepatitis B virus (HBV) infection is a major risk factor for HCC. There is mounting evidence to support a role for the hepatitis B virus X (HBx) gene and protein in the pathogenesis of HBV-induced HCC. HBx gene is often included, and remains functionally active, in the HBV DNA that is frequently integrated into cellular DNA during hepatocellular carcinogenesis. HBx protein has now been extensively shown to promote tumor growth and resistance to therapy in human HCC through modulating apoptosis, telomerase activity, nucleotide excision repair, cell cycle, cell adhesion, epigenetics, etc. More recent studies have also found HBx to induce tumorigenicity of hepatic progenitor cells in DDC-treated HBx transgenic mice [24], to inhibit cell differentiation in hepatic progenitor cells [39] and to play an anti-apoptosis role in hepatic progenitor cells by activating the Wnt/beta-catenin pathway [40]. HBx has also been shown to trigger HCC transformation by promoting properties that are characteristics of CSCs and EpCAM [41–42]. Our results is consistent with these reports where the presence of a stable and replicating HBV genome in the HepG2 culture system (HepG2.2.15) resulted in the enhanced ability of the cells to self-renew as demonstrated by spheroid formation assay and the induced expression of stemness related genes and markers (ABCB1, ABCC2, ALDH1A1, CD133, NANOG and SMO) at both genomic and proteomic levels.

Our group and others have previously reported that HBV integration is detected in 80–90% of host genomes from HBV-infected HCC cases and that the HBx gene is often partially deleted during the integration process, causing the C-terminal truncation of HBx [6–8]. We and others have also found C-terminal truncated HBx to play a critical pro-oncogenic and pro-metastatic role in hepatocarcinogenesis [10–14] through modulating GAS2 mediated deregulation of cell cycle and senescence, c-Jun/MMP10 and distinct miRNA transcription. However, whether C-terminal truncated HBx trigger HCC formation through induction of cancer stem cell-like features or a CSC subpopulation remains unknown.

Here in this study, we attempted to explore the role of C-terminal truncated HBx in induction of “stemness” phenotypic properties and induction of a CD133 subset. We chose the previously widely reported C-terminal truncated HBx mutants with a breakpoint at 140 aa (HBxΔC14) and breakpoint at 119 aa (HBxΔC35) for our cell model because these two mutants have been shown to abrogate the growth suppressive effects induced by full-length HBx, effectively promoting cell transformation and enhancing the proliferative activity of neoplastic cells [8, 11, 25, 29]. These two mutants were also selected because they have previously been identified as natural deleting mutants in HCC tissues [8, 25]. HBx-ΔC was exclusively detected in HCC cell lines that were raised from patients presented with a HBV background and with concomitant CD133 expression (Hep3B, PLC8024, SNU182 and SNU475). Both HBxΔC14 and HBxΔC35 also induced the appearance of a CD133 liver CSC subpopulation in MIHA cells, mediated via STAT3 activation. CD133 is a well-documented functional liver CSC marker, known to be present in HCC tumors and contribute to tumor recurrence and therapy resistance [16–22]. Recent study by Won et al. found CD133 transcription in HCC to be mediated via IL-6/STAT3 activation [35]. Interestingly, full-length HBx was previously found to induce EpCAM expression in HCC [23, 42], whose expression is known to overlap to some degree with CD133 [22]. We then went on to test the ability of MIHA and Huh7 cells stably overexpressed with empty vector control, HBxΔC14 and HBxΔC35 to confer various cancer and stem cell-like properties *in vitro*. As compared to the control, HBx-Δ14 or HBx-Δ35 resulted in a significant increase of the cells ability to self-renew, resist chemotherapy (5-FU) and the only targeted therapy approved for treatment of HCC (sorafenib), migrate and induce tube formation in endothelial cells. Thus, HBxΔC14 and HBxΔC35 showed more pronounced phenotypes consistent with stem cell behavior than EV control cells. Hence, these phenotypic characteristics presented were consistent with the induction of CD133 marker by C-terminal truncated HBx mutants.

To further dissect the mechanistic basis of the HBxΔC14 and HBxΔC35 mediated stemness, a global transcriptome sequencing profiling was employed to examine the altered profiles of C-terminal truncated mutants. HBxΔC14 and HBxΔC35 exhibit an enhanced expression of stemness gene signatures involved in FXR/RXR activation and drug metabolism, including ABC transporters (ABCG2, ABCB1 and ABCB4), solute carrier family members (SLCO1B3 and SLC51A/OSTA), cytosolic glycosyltransferase (UGT1A1, UGT2B11 and UGT2B4), homeostasis-related genes (LIPC, VLDLR, PPARG, PGC1A, HPX), apolipoproteins (APOC3, APOA4 and APOA1), as well as FGA, CETP, GSTM2, C4B, AGT, SAA4, PKLR, FETUB, IL18, DPYD and CREBBP/CBP. This is consistent with past studies where activation of the nuclear receptor FXR enhances hepatocyte chemoprotection and liver tumor chemoresistance against genotoxic compounds [37–38]; and the link between ABCG2 and resistance to chemotherapy in HCC [43]. Subsequent rescue experiments with a selective FXR inhibitor further validated this bioinformatics prediction, suggesting that C-terminal truncated HBx can mediate cancer and stemness features via FXR activation.

To conclude, our data suggest that C-terminal truncated HBx, in particular at 140 aa and 119 aa breakpoints, enhances stemness properties *in vitro* and induces a CD133 liver CSC subpopulation in HCC through modulating a distinct altered genomic profile involving FXR pathway and possibly drug metabolism (Figure 5C).

## MATERIALS AND METHODS

### Reagents

Sorafenib was purchased from LC Laboratories. FXR inhibitor, Z-guggulsterone was purchased from Tocris Bioscience and used at 25 μM.

### Cell lines

Human HCC cell lines Hep3B, SNU-182, SNU-475 and HepG2, were purchased from American Type Culture Collect (ATCC). Human liver cell line LO2 and HCC cell lines PLC8024, QSG-7701 and BEL7402, were obtained from the Institute of Virology, Chinese Academy of Medical Sciences, Beijing, China. HCC cell line, Huh7, was provided by Dr. H. Nakabayashi, Hokkaido University School of Medicine, Japan. H2P and H2M were previously established in our laboratory [34]. Immortalized normal liver cell line, MIHA, was provided by Dr. J. R. Chowdhury, Albert Einstein College of Medicine, New York. Human umbilical vein endothelial cells (HUVEC) were purchased from Invitrogen. HepG2.2.15 cells were cultured in DMEM supplemented with 10% fetal calf serum, 2 mM L-glutamine and 380 μg/mL G418. All cell lines used in this study were regularly authenticated by morphological observation and tested for absence of Mycoplasma contamination (MycoAlert, Lonza).

### Flow cytometry and cell sorting

Flow cytometry analysis or flow cytometry cell sorting was conducted using PE-conjugated mouse anti-human CD133 (Miltenyi Biotec) or its respective isotype control. ALDEFLUOR reagent (Stem Cell Technologies) was used for the immunofluorescent detection of intracellular ALDH enzyme activity. Samples were analyzed on BD FACSCanto II (BD Biosciences) with data analyzed by FlowJo (Tree Star Inc.).

### Detection of full-length and C-terminal truncated HBx by RT-PCR

RT-PCR was performed in matched non-tumor and HCC tissue samples using primers encompassing the entire and different lengths of X gene, as previously described [10]. Specifically, for PCR amplification of HBx, sets of PCR primers (44F: 5′-TCCTTTGTTTACGTCCCGTC-3′, XR1: 5′-GCAGATGAGAAGGCACAGAC-3′ and 465R: 5′-TTAGGCAGAGGTGAAAAAGTTGC-3′) were used for full-length and C-terminal truncated HBx, respectively. Clones that can only be amplified by the primers for the smaller fragment (flanking 197nt), but not for the full-length fragment (flanking 465nt), was considered positive for C-terminal truncated HBx. RT-PCR products were analyzed by electrophoresis on a 1.5% agarose gel, stained with ethidium bromide and visualized on a Gel Doc XR System (Bio-Rad).

### Quantitative real-time PCR

Total RNA was extracted using RNA-IsoPlus (Takara) and cDNA was synthesized by PrimeScript RT Master Mix (Takara). qRT-PCR was performed with SYBR Green PCR Master Mix (Applied Biosystems) and primers as listed in Supplementary Table 1 on an ABI Prism 7900 System with data analyzed using the ABI SDS v2.3 software (Applied Biosystems). Relative expression differences were calculated using the 2^−ΔΔCt^ method.

### Western blot

Protein lysates were quantified and resolved on a SDS-PAGE gel, transferred onto a PVDF membrane (Millipore) and immunoblotted with a primary antibody, followed by incubation with a secondary antibody. Antibody signal was detected using an enhanced chemiluminescence system (GE Healthcare). The following antibodies were used: FLAG (M2) (1:2000, Sigma-Aldrich, F3165), p-STAT3 (1:1000, Cell Signaling, 9145S), total STAT3 (1:1000, Cell Signaling, 9132) and β-actin (1:5000, Sigma-Aldrich, A5316).

### Establishment of C-terminal truncated HBx stably expressing cell lines

Viral DNA samples extracted from the sear of HCC patient CH230 were used for the amplification and cloning of HBx gene as previously described [11]. HBx fragments from patient CH230 were amplified by PCR using a forward primer carrying Kozak and flag-tag sequence and reverse primers with an artificial stop codon at different deletion sites. PCR products were cloned into the HindIII and EcoR1 restriction sites of a pcDNA6b vector to generate flag-tagged HBxΔ14 and HBxΔ35, expressing 14- and 35- aa carboxyl-terminal truncation, respectively. Huh7 and MIHA cells were transfected using Lipofectamine 2000 (Invitrogen) with stable expressing pooled clones selected using blasticidin at a concentration of 5 μg/mL. Empty vector pcDNA6b-3xFLAG was also transfected into Huh7 and MIHA cells as negative controls.

### Hepatosphere-forming and self-renewal assay

Single cells were cultured in 300 μl of serum-free DMEM/F12 medium (Invitrogen) supplemented with 20 ng/ml human recombinant epidermal growth factor (Sigma-Aldrich), 10 ng/ml human recombinant basic fibroblast growth factor (Sigma-Aldrich), 4 μg/ml insulin (Sigma-Aldrich), B27 (1:50; Invitrogen), 500 U/ml penicillin, 500 μg/ml streptomycin (Invitrogen) and 1% methylcellulose (Sigma-Aldrich). Cells were cultured in suspension in poly-HEMA-coated 24-well plates. Cells were replenished with 30 μl of supplemented medium every second day. To propagate spheres *in vitro*, spheres were collected by gentle centrifugation and dissociated to single cells using TrypLE Express (Invitrogen). Following dissociation, trypsin inhibitor (Invitrogen) was used to neutralize the reaction, and the cells were cultured to generate the next generation of spheres.

### Cell motility assay

Migration assays were conducted in 24-well Millicell hanging inserts (Millipore). Cells re-suspended in serum free DMEM were added to the top chamber and medium supplemented with 10% FBS was added to the bottom chamber as a chemoattractant. After 48 hrs of incubation at 37°C, cells that migrated through the membrane were fixed and stained with crystal violet (Sigma-Aldrich). The number of cells was counted in 3 random fields under 20x objective lens and imaged using SPOT imaging software (Nikon).

### Annexin V apoptosis assay

Cells were treated with 5-FU (Huh7 with 300 μg/mL and MIHA with 25 μg/mL) or sorafenib (Huh7 with 5 μM and MIHA with 2 μM) for 48 hrs. Following treatment, cells were harvested and stained with propidium iodide (PI) and FITC-conjugated Annexin V as provided by the Annexin V-FLUOS Staining Kit (Roche). Samples were analyzed on BD FACSCanto II (BD Biosciences) with data analyzed by FlowJo (Tree Star Inc.).

### Capillary tube formation assay in HUVECs

Cells were cultured in 6-well plates in complete medium, with culture medium replaced with serum free medium after 24 hrs. Conditioned medium was collected and filtered after incubation for a further 24 hrs. HUVECs were co-cultured with conditioned medium for 24 hrs. Capillary tube formation assays were then conducted on BD Matrigel Basement Membrane Matrix (BD Biosciences). The number of capillary tubes formed was counted in 3 random fields under a 20x objective lens and imaged using SPOT imaging software. Specifically, the formation of 1 capillary tube was defined as a connection between 2 cells.

### RNA sequencing, pre-processing, alignment and expression quantification

RNA-Seq was performed as a service at the Centre for Genomic Sciences of The University of Hong Kong using the Illumina HiSeq platform. cDNA libraries were prepared using the KAPA Stranded mRNA-Seq Kit using 1 μg of total RNA. HiSeq PE Cluster Kit v4 with cbot was used for cluster generation on the flow cell. Illumina HiSeq SBS Kit v4 was used for pair-end 101bp sequencing. To obtain high quality reads for downstream analysis, raw sequencing reads were first filtered for adapter sequences and low quality (reads containing unknown bases and bases having a quality value of ≤ 10) and removed using cutadapt [44] and custom scripts bases. Low complexity bases (more than 10bp of continuous bases of “A” and “T”) were trimmed from the 3' end using prinseq [45]. Reads with length < 40bp were then discarded. Sequencing reads were also filtered for rRNA sequence and remaining high quality reads were used for downstream analysis. High quality reads were aligned against human transcriptome (hg19) using RSEM package v1.2.21 [46]. Expression estimation and tests for differential expression were performed using EBSeq v1.6.0 in the RSEM package [47]. Transcriptome sequencing data available publicly at Gene Expression Omnibus (http://www.ncbi.nlm.nih.gov/geo/) under accession number GSE71993.

### Pathway analysis

RNA sequencing data were formatted and uploaded to the IPA software (Ingenuity Systems). Filter parameters (fold change ≥ 1.5 with FDR ≤ 0.05) for deregulated genes were set before running analysis. Top 10 results for core analysis on biological and toxicological functions, and canonical pathways were sorted according to the *p*-value. Fisher's exact test was used to estimate the probability of association between a set of molecules and a function or pathway. The pathway/network shows the molecular relationship between molecules based on Ingenuity Knowledge Database.

### Statistical analysis

Statistical analyses were performed using GraphPad Prism 5.0 (GraphPad Software, Inc.) and SPSS version 21.0 (IBM). Independent Student's *t*-test was used to compare the mean value of two groups. Error bars represent SD values. Statistical significance was defined as *p* ≤ 0.05.

## SUPPLEMENTARY MATERIALS TABLES



## References

[1] Tanaka M, Katayama F, Kato H, Tanaka H, Wang J, Qiao YL, Inoue M (2011). Hepatitis B and C virus infection and hepatocellular carcinoma in China: a review of epidemiology and control measures. J Epidemiol.

[2] Matsubara K, Tokino T (1990). Integration of HBV DNA and its implications for hepatocarcinogenesis. Mol Biol Med.

[3] Feitelson MA, Duan LX (1997). Hepatitis B virus X antigen in the pathogenesis of chronic infections and the development of hepatocellular carcinoma. Am J Pathol.

[4] Takada S, Koike K (1990). Trans-activation function of a 3' truncated X gene-cell fusion product from integrated HBV DNA in chronic hepatitis tissues. Proc Natl Acad Sci USA.

[5] Diamantis ID, McGandy CE, Chen TJ, Liaw YF, Gudat F, Bianchi L (1992). Hepatitis B X-gene expression in hepatocellular carcinoma. J Hepatol.

[6] Wang Y, Wu MC, Sham JS, Tai LS, Fang Y, Wu WQ, Xie D, Guan XY (2002). Different expression of hepatitis B surface antigen between hepatocellular carcinoma and its surrounding liver tissue detected by tissue microarray. J Pathol.

[7] Wang Y, Lau SH, Sham JS, Wu MC, Wang T, Guan XY (2004). Characterization of HBV integrants in 14 hepatocellular carcinomas: association of truncated X gene and hepatocellular carcinogenesis. Oncogene.

[8] Tu H, Bonura C, Giannini C, Mouly H, Soussan P, Kew M, Paterlini-Brechot P, Brechot C, Kremsdorf D (2001). Biological impact of natural COOH-terminal deletions of hepatitis B virus X protein in hepatocellular carcinoma tissues. Cancer Res.

[9] Liu XH, Lin J, Zhang SH, Zhang SM, Feitelson MA, Gao HJ, Zhu MH (2008). COOH-terminal deletion of HBx gene is a frequent event in HBV-associated hepatocellular carcinoma. World J Gastroenterol.

[10] Ma NF, Lau SH, Hu L, Xie D, Wu J, Yang J, Wang Y, Wu MC, Fung J, Bai X, Tzang CH, Fu L, Yang M (2008). COOH-terminal truncated HBV X protein plays key role in hepatocarcinogenesis. Clin Cancer Res.

[11] Yip WK, Cheng AS, Zhu R, Lung RW, Tsang DP, Lau SS, Chen Y, Sung JG, Lai PB, Ng EK, Yu J, Wong N, To KF (2011). Carboxyl-terminal truncated HBx regulates a distinct microRNA transcription program in hepatocellular carcinoma development. PLoS One.

[12] Zhu R, Mok MT, Kang W, Lau SS, Yip WK, Chen Y, Lai PB, Wong VW, To KF, Sung JJ, Cheng AS, Chan HL (2015). Truncated HBx-dependent silencing of GAS2 promotes hepatocarcinogenesis through deregulation of cell cycle, senescence and p53-mediated apoptosis. J Pathol.

[13] Sze KM, Chu GK, Lee JM, Ng IO (2013). C-terminal truncated hepatitis B virus x protein is associated with metastasis and enhances invasiveness by C-Jun/matrix metalloproteinase protein 10 activation in hepatocellular carcinoma. Hepatology.

[14] Quetier I, Brezillon N, Revaud J, Ahodantin J, DaSilva L, Soussan P, Kremsdorf D (2015). C-terminal-truncated hepatitis B virus X protein enhances the development of diethylnitrosamine-induced hepatocellular carcinogenesis. J Gen Virol.

[15] Marquardt JU, Thorgeirsson SS (2010). Stem cells in hepatocarcinogenesis: evidence from genomic data. Semin Liver Dis.

[16] Ma S, Chan KW, Hu L, Lee TK, Wo JY, Ng IO, Zheng BJ, Guan XY (2007). Identification and characterization of tumorigenic liver cancer stem/progenitor cells. Gastroenterology.

[17] Ma S, Tang KH, Chan YP, Lee TK, Kwan PS, Castilho A, Ng IO, Man K, Wong N, To KF, Zheng BJ, Lai PB, Lo CM (2010). miR-130b promotes CD133(+) liver tumor-initiating cell growth and self-renewal via tumor protein 53-induced nuclear protein 1. Cell Stem Cell.

[18] Suetsugu A, Nagaki M, Aoki H, Motohashi T, Kunisada T, Moriwaki H (2006). Characterization of CD133+ hepatocellular carcinoma cells as cancer stem/progenitor cells. Biochem Biophys Res Commun.

[19] Yin S, Li J, Hu C, Chen X, Yao M, Yan M, Jiang G, Ge C, Xie H, Wan D, Yang S, Zheng S, Gu J (2007). CD133 positive hepatocellular carcinoma cells possess high capacity for tumorigenicity. Int J Cancer.

[20] Ma S, Chan KW, Lee TK, Tang KH, Wo JY, Zheng BJ, Guan XY (2008). Aldehyde dehydrogenase discriminates CD133 liver cancer stem cell populations. Mol Cancer Res.

[21] Ma S, Lee TK, Zheng BJ, Chan KW, Guan XY (2008). CD133+ HCC cancer stem cells confer chemoresistance by preferential expression of the Akt/PKB survival pathway. Oncogene.

[22] Tang KH, Ma S, Lee TK, Chan YP, Kwan PS, Tong CM, Ng IO, Man K, To KF, Lai PB, Lo CM, Guan XY, Chan KW (2012). CD133(+) liver tumor-initiating cells promote tumor angiogenesis, growth, and self-renewal through neurotensin/interleukin-8/CXCL1 signaling. Hepatology.

[23] Arzumanyan A, Friedman T, Ng IO, Clayton MM, Lian Z, Feitelson MA (2011). Does the hepatitis B antigen HBx promote the appearance of liver cancer stem cells?. Cancer Res.

[24] Wang C, Yang W, Yan HX, Luo T, Zhang J, Tang L, Wu FQ, Zhang HL, Yu LX, Zheng LY, Li YQ, Dong W, He YQ (2012). Hepatitis B virus X (HBx) induces tumorigenicity of hepatic progenitor cells in 3,5-diethoxycarbonyl-1,4-dihydrocollidine-treated HBx transgenic mice. Hepatology.

[25] Xu R, Zhang X, Zhang W, Fant Y, Zheng S, Yu XF (2007). Association of human APOBEC3 cytidine deaminases with the generation of hepatitis virus B x antigen mutants and hepatocellular carcinoma. Hepatology.

[26] Sells MA, Chen ML, Acs G (1987). Production of hepatitis B virus particles in HepG2 cells transfected with cloned hepatitis B virus DNA. Proc Natl Acad Sci USA.

[27] Cheng AS, Wong N, Tse AM, Chan KY, Chan KK, Sung JJ, Chan HL (2007). RNA interference targeting HBx suppresses tumor growth and enhances cisplatin chemosensitivity in human hepatocellular carcinoma. Cancer Lett.

[28] Chan DW, Ng IO (2006). Knock-down of hepatitis B virus X protein reduces the tumorigenicity of hepatocellular carcinoma cells. J Pathol.

[29] Li CH, Xu F, Chow S, Feng L, Yin D, Ng TB, Chen Y (2014). Hepatitis B virus X protein promotes hepatocellular carcinoma transformation through interleukin-6 activation of microRNA-21 expression. Eur J Cancer.

[30] Edman JC, Gray P, Valenzuela P, Rall LB, Rutter WJ (1980). Integration of hepatitis B virus sequences and their expression in a human hepatoma cell. Nature.

[31] Twist EM, Clark HF, Aden DP, Knowles BB, Plotkin SA (1981). Integration pattern of hepatitis B virus DNA sequences in human hepatoma cell lines. J Virol.

[32] Knowles BB, Searls DV, Aden DP Advances in Hepatitis Research (FV, editor).

[33] Ku JL, Park JG (2005). Biology of SNU cell lines. Cancer Res Treat.

[34] Hu L, Wen JM, Sham JS, Wang W, Xie D, Tjia VM, Huang JF, Zhang M, Zeng WF, Guan XY (2004). Establishment of cell lines from a primary hepatocellular carcinoma and its metastasis. Cancer Genet Cytogent.

[35] Won C, Kim BH, Yi EH, Choi KJ, Kim EK, Jeong JM, Lee JH, Jang JJ, Yoon JH, Jeong WI, Park IC, Kim TW, Bae SS (2015). Signal transducer and activator of transcription 3-mediated CD133 up-regulation contributes to promotion of hepatocellular carcinoma. Hepatology.

[36] Herraez E, Gonzalez-Sanchez E, Vaquero J, Romero MR, Serrano MA, Marin JJ, Briz O (2012). Cisplatin-induced chemoresistance in colon cancer cells involves FXR-dependent and FXR-independent up-regulation of ABC proteins. Mol Pharm.

[37] Vaquero J, Briz O, Herraez E, Muntane J, Marin JJ (2013). Activation of the nuclear receptor FXR enhances hepatocyte chemoprotection and liver tumor chemoresistance against genotoxic compounds. Biochim Biophys Acta.

[38] Ohno T, Shirakami Y, Shimizu M, Kubota M, Sakai H, Yasuda Y, Kochi T, Tsurumi H, Moriwaki H (2012). Synergistic growth inhibition of human hepatocellular carcinoma cells by acyclic retinoid and GW4064, a farnesoid X receptor ligand. Cancer Lett.

[39] Huang J, Shen L, Lu Y, Li H, Zhang X, Hu D, Feng T, Song F (2012). Parallel induction of cell proliferation and inhibition of cell differentiation in hepatic progenitor cells by hepatitis B virus X gene. Int J Mol Med.

[40] Shen L, Zhang X, Hu D, Feng T, Li H, Lu Y, Huang J (2013). Hepatitis B virus X (HBx) play an anti-apoptosis role in hepatic progenitor cells by activating Wnt/β-catenin pathway. Mol Cell Biochem.

[41] Arzumanyan A, Sambandam V, Clayton MM, Choi SS, Xie G, Diehl AM, Yu DY, Feitelson MA (2012). Hedgehog signaling blockade delays hepatocarcinogenesis induced by hepatitis B virus X protein. Cancer Res.

[42] Fan H, Zhang H, Pascuzzi PE, Andrisani O (2016). Hepatitis B virus X protein induces EpCAM expression via active DNA demethylation directed by RelA in complex EZH2 and TET2. Oncogene.

[43] Wang XQ, Ongkeko WM, Chen L, Yang ZF, Lu P, Chen KK, Lopez JP, Poon RT, Fan ST (2010). Octamer 4 (Oct4) mediates chemotherapeutic drug resistance in liver cancer cells through a potential Oct4-AKT-ATP-binding cassette G2 pathway. Hepatology.

[44] Martin M (2011). Cutadapt removes adapter sequences from high-throughput sequencing reads. EMBnet J.

[45] Schmieder R, Edwards R (2011). Quality control and preprocessing of metagenomics datasets. Bioinformatics.

[46] Li B, Dewey CN (2011). RSEM: accurate transcript quantification from RNA-Seq data with or without a reference genome. BMC Bioinformatics.

[47] Leng N, Dawson JA, Thomsom JA, Ruotti V, Rissman AI, Smits BM, Haag JD, Gould MN, Stewart RM, Kendziorski C (2013). EBSeq: an empirical Bayes hierarchical model for interference in RNA-seq experiments. Bioinformatics.

