# Supplementary material for: C-terminal truncated hepatitis B virus X protein promotes hepatocellular carcinogenesis through induction of cancer and stem cell-like properties
Source: Oncotarget. 2016 Mar 19;7(17):24005–17. doi: 10.18632/oncotarget.8209 (PMC5029680; doi:10.18632/oncotarget.8209)
Supplement: Supplementary file 1 [file oncotarget-07-24005-s001.pdf]

C-terminal truncated hepatitis B virus X protein promotes hepatocellular carcinogenesis through induction of cancer and stem cell-like properties

Supplementary Materials

Supplementary Table S1: qRT-PCR primers used in this study

Gene	Forward Primer (5′–3′)	Reverse Primer (5′–3′)	GenBank No.
β-actin	CATCCACGAAACTACCTTCAACTCC	GAGCCGCCGATCCACACG	NM_001101
SLCO1B3	TGGAGCAACAGTACGGTCAG	TGCTTTCGCAGATTAGAGGGAA	NM_019844
CETP	GGCCAAGTCAAGTATGGGTTG	ACAGACACGTTCTGAATGGAGA	NM_000078
UGT2B4	GTATTGGCATCTTCAGCTTCCATTTC	AAGTTCTGCCCATCTCTTAACCAGC	NM_021139
ABCB4	ATAGCTCACGGATCAGGTCTC	GGATTTAGCAGCGACAAGGAAA	NM_018850
LIPC	ATCAAGTGCCCTTGGACAAAG	TGACAGCCCTGATTGGTTTCT	NM_000236
SLC51A	AAGAAGGCGTATTGGAAAGGG	ACCTCGTTTTATGCCGTGTG	NM_152672
VLDLR	AGAAAAGCCAAATGTGAACCCT	CACTGCCGTCAACACAGTCT	NM_001018056
FETUB	CCATGTGCTCAGAAAGAAGGC	ACTGGGCGAAGAGTACAGTTA	NM_014375
HPX	CGTGACTGAACGCTGCTCA	CTCCCGGTCCCATTTGTGAC	NM_000613
PPARG	ACCAAAGTGCAATCAAAGTGGA	ATGAGGGAGTTGGAAGGCTCT	NM_138711
IL18	TCTTCATTGACCAAGGAAATCGG	TCCGGGGTGCATTATCTCTAC	NM_001562
PGC1A	TCAGTCCTCACTGGTGGACA	AGGCAGCCCTCTGCTTCAGTGA	NM_013261
CREBBP/CBP	CAACCCCAAAAGAGCCAAACT	CCTCGTAGAAGCTCCGACAGT	NM_004380
UGT1A1	CTGTCTCTGCCCACTGTATTCT	TCTGTGAAAAGGCAATGAGCAT	NM_000463
DPYD	GGCGGACATCGAGAGTATCCT	TTCTTGGCCGAAGTGGAACAC	NM_000110
ABCB1	AAATTGGCTTGACAAGTTGTATATGG	CACCAGCATCATGAGAGGAAGTC	NM_000927
ABCC2	ATGCAGCCTCCATAACCATGA	CTTCGTCTTCCTTCAGGCTATTCA	NM_000392
ALDH1A1	CGCAAGACAGGCTTTTCAG	TGTATAATAGTCGCCCCCTCTC	NM_000689
CD133	TGGATGCAGAACTTGACAACGT	ATACCTGCTACGACAGTCGTGGT	NM_006017
NANOG	AATACCTCAGCCTCCAGCAGATG	TGCGTCACACCATTGCTATTCTTC	NM_024865
SMO	TGGTCACTCCCCTTTGTCCTCAC	GCACGGTATCGGTAGTTCTTGTAGC	NM_005631
SOX2	AAATGGGAGGGGTGCAAAAGAGGAG	CAGCTGTCATTTGCTGTGGGTGATG	NM_003106
